# Macular Ischemia Changes in Patients with Diabetic Macular Edema Treated with Aflibercept and Ranibizumab

**DOI:** 10.3390/diagnostics14121306

**Published:** 2024-06-20

**Authors:** Dimitrios Maris, Anna Dastiridou, Maria Kotoula, Aikaterini Karathanou, Evangelia E. Tsironi, Alexandra Bargiota, Sofia Androudi

**Affiliations:** 1 Ophthalmology Clinic, University of Thessaly, 41335 Larissa, Greece; d8maris@gmail.com (D.M.); adastirid@uth.gr (A.D.); mariarkotoula@gmail.com (M.K.); kathykarathanou@yahoo.gr (A.K.); e_tsironi@hotmail.com (E.E.T.); 2 Endocrinology Clinic, University of Thessaly, 41335 Larissa, Greece; abargio@med.uth.gr

**Keywords:** diabetic macular edema, ranibizumab, aflibercept, ischemia, fovea avascular zone, OCT angiography

## Abstract

Τhis study aims to assess changes in the fovea avascular zone (FAZ) in treatment naïve patients receiving aflibercept or ranibizumab injections for diabetic macular edema (DME). Best corrected visual acuity (BCVA) testing, OCT, and OCT-angiography imaging were performed at baseline and 1 month after each injection. Injections of either aflibercept or ranibizumab were administered monthly for 6 consecutive months. FAZ in the superficial (SCP) and the deep capillary plexus (DCP) using OCT angiography was recorded for each visit. Fifty eyes from fifty patients with a mean age of 67.0 ± 10.7 years were included in the study. Twenty-five patients received aflibercept and twenty-five received ranibizumab. BCVA was 40.8 ± 10.0 and increased to 52.1 ± 7.9 ETDRS letters at the last visit (*p* < 0.001). CRT was 295.6 ± 34.0 at baseline and 247.9 ± 29.7 at the last study visit (*p* < 0.001). SCP FAZ was 350.6 ± 79.5 μm^2^ at baseline and 339.0 ± 71.3 μm^2^ after sox monthly injections (*p* = 0.132). DCP FAZ was 558.6 ± 199.0 μm^2^ at baseline and 459.5 ± 156.1 μm^2^ after six monthly injections (*p* < 0.001). There was no effect of the choice of ranibizumab or aflibercept on DCP FAZ change (*p* = 0.277). In conclusion, treatment with 6 monthly injections of ranibizumab and aflibercept led to an increase in BCVA and a decrease in CRT and DCP FAZ area. Both drugs led to an improvement in DCP ischemia.

## 1. Introduction

Diabetic retinopathy is a major source of visual disability [1]. Diabetic macular ischemia is one of the vision-threatening complications of diabetic retinopathy. In macular ischemia, the structure of the foveal capillary network is damaged. Such damage includes the enlargement and irregularity of the foveal avascular zone (FAZ) and macular non-perfusion, leading to macular dysfunction. Fluorescein angiography (FA) is a well-established modality to assess perfusion of the retina and the level of ischemia in the macula and the peripheral retina.

The sequelae for the visual function of diabetic macular ischemia severity are well characterized since the Early-Treatment Diabetic Retinopathy Study (ETDRS) in the 1980s [2]. Diabetic macular ischemia is traditionally defined based on the evidence of an enlarged and irregular FAZ area of at least 0.5 mm^2^ or parafoveal capillary dropout in at least one parafoveal quadrant if the FAZ area is less than 0.5 mm^2^ on FA [2,3]. It is also understood that the magnitude of ischemic changes in the retina is more pronounced in the proliferative stage of diabetic retinopathy [4].

OCT-angiography is a new imaging modality that has addressed several drawbacks of FA, including limited image resolution, invasiveness, lack of depth information, and two-dimensional imaging [5,6]. OCT angiography provides depth-encoded imaging with improved resolution. It is now possible to visualize not only the superficial (SCP), but also the deep (DCP) capillary plexus of the retina, to non-invasively quantify several FAZ parameters and to assess separately the integrity of each capillary plexus [7,8,9]. OCT angiography images offer both qualitative as well as quantitative information. However, the association between the microvascular changes that can be detected in OCT angiography in diabetic macular ischemia and visual impairment is complex and largely unknown, while recent studies are trying to better characterize this relationship [3,10]. The FAZ area size has been one of the biomarkers that have been associated with varying levels of retinopathy severity [11]. Moreover, a larger baseline deep capillary plexus fovea avascular zone (DCP FAZ) area has been reported to be predictive of worsening visual outcomes [12]. 

Intravitreal injections of anti-vascular endothelial growth factor (VEGF) are the preferred initial treatment approach for diabetic macular edema (DME) due to their proven safety and effectiveness [13]. These drugs selectively block VEGF, which is regarded as a key mediator in diabetic retinopathy. Notably, as the VEGF counteracts the blockage of the foveal capillary network, which causes diabetic macular ischemia, administering anti-VEGF injections should be advantageous for diabetic macular ischemia. Furthermore, current research is actively attempting to assess the biomarkers from OCT and OCT angiography imaging that play a crucial role in predicting the visual outcome following intravitreal anti-VEGF injections [14]. A larger DCP FAZ area has emerged as one of these factors [15].

OCT angiography additionally offers a novel instrument for evaluating the impact of treatment [16]. The objective of this pilot study was to investigate the alteration in the area of the FAZ following the administration of intravitreal anti-VEGF injections. Our objective was to examine whether there is any variation in the treatment effect between ranibizumab and aflibercept, as well as to investigate the relationship between macular ischemia and visual acuity during the treatment process.

## 2. Materials and Methods

Treatment naive patients diagnosed with center-involved diabetic macular edema were consecutively enrolled in the Retina clinics at the University Hospital of Larissa, Greece, between June 2017 and June 2022. This was a single-center prospective study. The allocation of patients to receive 0.05 mL of either ranibizumab 10 mg/mL or aflibercept 40 mg/mL was determined by the treating physician. The study protocol adhered to the Declaration of Helsinki and was approved by the University Hospital of Larissa Review Board. Before enrolling, eligible participants were required to sign an informed consent form and were provided with a thorough explanation of the study protocol.

The inclusion criteria were as follows: age of 18 years or older, the presence of type 1 or 2 diabetes, treated with insulin or oral anti-hyperglycemia agents, the presence of center-involved DME with central subfield thickness measuring between 280 and 600 microns, and best-corrected visual acuity (BCVA) Early Treatment Diabetic Retinopathy Study (ETDRS) score between 73 and 24 letters (equivalent to 20/40–20/320 decimal visual acuity) in the study eye. Patients were recruited in a consecutive if eligible fashion.

Patients who met any of the following criteria were not included in the study: poor glycemic control (hemoglobin HbA1c > 8 in the past 6 months), uncontrolled arterial hypertension, active proliferative diabetic retinopathy (PDR), or history of vitreous hemorrhage, prior panretinal photocoagulation, concomitant ocular conditions (other than diabetic retinopathy) that could affect visual acuity or imaging (e.g., retinal vein or artery occlusion, cornea scarring, dense cataract, uveitis, or other ocular inflammatory disease, glaucoma, vitreomacular traction, epiretinal membrane, etc.), previous vitrectomy and prior ocular surgeries (other than uneventful cataract extraction, performed at least three months before enrollment). Only treatment-naive patients were included in the study; therefore, patients who had previously received intravitreal anti-VEGF or corticosteroid injections or macula laser photocoagulation were excluded. Patients that would develop vitreous hemorrhage or necessitate rescue panretinal photocoagulation would exit the study. All patients were offered treatment with monthly injections up to 6 months. All injections were administered in time intervals of 30 ± 3 days. Each visit included the BCVA measurement assessed with an ETDRS chart, slit lamp examination and Goldmann applanation tonometry, followed by OCT and OCT angiography imaging. At the end of the baseline visit, the first intravitreal injection of ranibizumab or aflibercept was given. Then, on visit 1, after the clinical examination and imaging sessions, the second injection was delivered. Likewise, visit 2 ended with the 3rd intravitreal injection, visit 3 with the 4th injection, visit 4 with the 5th injection, visit 5 with the 6th injection, and visit 6 was scheduled at 1 month after the 6th injection. OCT and OCT angiography imaging were performed using the Triton Plus swept source OCT (Topcon Inc., Tokyo, Japan). Scanning protocols included a 6 × 6 mm 3D OCT angiography scan with a resolution of 320 × 320 pixels and 3D OCT 7 × 7 mm scan with a resolution of 512 × 256 pixels. OCT central retina thickness (CRT) was quantified with the instrument’s built-in software. The automatic segmentation algorithms of the OCT angiography device were used. The SCP slab spanned the area between the inner limiting membrane (ILM) + 2.6 μm and an outer boundary at the inner plexiform layer (IPL)/inner nuclear layer (INL) + 15.6 μm. The DCP slab was segmented between IPL/INL + 15.6 μm and IPL/INL + 70.2 μm.

En face OCT angiography images of the SCP and DCP were analyzed using the instrument’s built-in tool to quantify the area of the SCP and DCP FAZ area by two graders (DM and AD). The mean of the two measurements was recorded for each image. All scans were acquired by one grader (DM). Images were visually inspected for artifacts, motion artifacts, and low signal strength. Images with a low signal strength (less than 70), artifacts and segmentation errors were rejected and the scan was repeated.

All statistical testing was performed using the SPSS 23 statistical package (IBM Inc, Armonk, NY, USA). Continuous variables were tested for normality and data were presented as the mean and standard deviation or median and interquartile range accordingly. Repeated measurements of the outcome variables at specific timepoints over time were evaluated with repeated measures ANOVA and the Wilks’ Lambda test. Estimates of the effect size and observed power were also calculated. Post hoc tests were used with the corresponding *p*-values to evaluate differences between individual visits. The amount of change in the SCP FAZ area as well as DCP FAZ area was also calculated and compared between consecutive visits with repeated measures ANOVA. An independent samples *t*-test was used to compare between groups and the chi-square test was used to compare categorical variables. Furthermore, Pearson correlation tests were performed. The level of significance was set at 0.05.

## 3. Results

Fifty eyes with full sets of data were analyzed. The mean age was 67.0 ± 10.7 years. There were 25 men and 25 women. Twenty-five patients received monthly aflibercept and the other twenty-five received monthly ranibizumab for 6 consecutive months. There was no difference in age (68.7 ± 7.9 versus 64.4 ± 12.0, independent samples *t*-test, *p* = 0.067) or gender (11 men/14 women vs. 14 men/11 women, chi-square *p* = 0.572) between the two groups. None of the patients developed vitreous hemorrhage or required laser photocoagulation during the study. None of the patients was lost to follow up.

**Figure 1:**
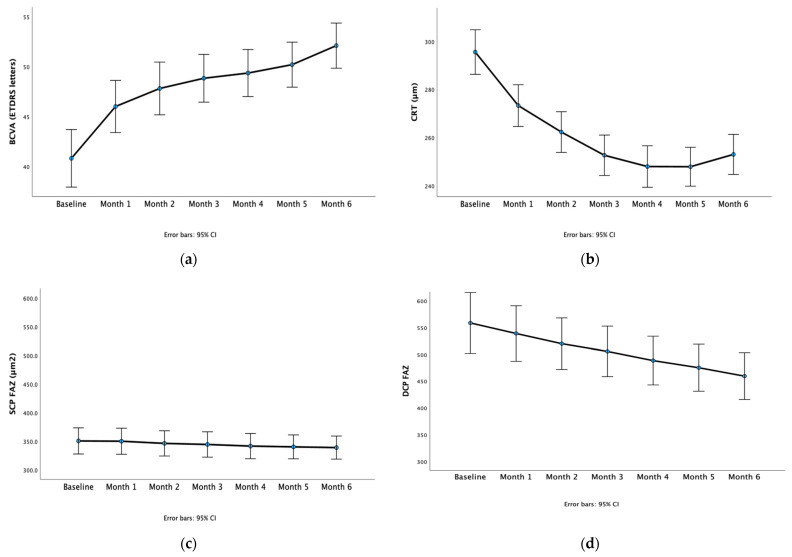
Line graphs of the measurements at baseline and each monthly visit of best corrected visual acuity (BCVA, (**a**)), central retina thickness (CRT, (**b**)), superficial capillary plexus fovea avascular zone area (SCP FAZ, (**c**)), and deep capillary plexus fovea avascular zone area (DCP FAZ, (**d**)). Error bars represent 95% confidence intervals.

BCVA was 40.8 ± 10.0 ETDRS letters and increased to 52.1 ± 7.9 ETDRS letters at the last visit (Table 1, Figure 1a). There was significant improvement in BCVA over the course of the study (*p* < 0.001). Post hoc pairwise comparisons showed a significant increase in BCVA in all study visits compared to the baseline (all *p* < 0.001). No effect of the choice of drug being either ranibizumab or aflibercept on BCVA results was found (*p* = 0.428).

CRT was 295.6 ± 34.0 at baseline and 247.9 ± 29.7 at the last study visit (Table 1, Figure 1b). CRT decreased with the treatment (*p* < 0.001). Post hoc pairwise comparisons showed that the CRT measurements at each subsequent study visit were lower compared to baseline (all *p* < 0.001). There was no effect of the type of treatment (ranibizumab or aflibercept) on the amount of CRT change (*p* = 0.261).

SCP FAZ area was 350.6 ± 79.5 μm^2^ at baseline and 339.0 ± 71.3 μm^2^ after 6 monthly injections (Table 1, Figure 1c). In visit 1, the SCP FAZ area was 350.1 ± 79.2 μm^2^; in visit 2, it was 346.3 ± 77.0 μm^2^; in visit 3, it was 344.4 ± 77.6 μm^2^; in visit 4, it was 341.6 ± 77.6 μm^2^; and in visit 5, it measured 340.2 ± 73.3 μm^2^. There was no significant change in the SCP FAZ area when comparing the SCP FAZ area at the seven study timepoints (*p* = 0.132) or between the two drugs (*p* = 0.092).

The DCP FAZ area was 558.6 ± 199.0 μm^2^ at baseline and 459.5 ± 156.1 μm^2^ after six monthly injections (*p* < 0.001, Table 1, Figure 1d). In visit 1, the DCP FAZ area was 539.0 ± 182.0 μm^2^; in visit 2, it was 520.0 ± 169.4 μm^2^; in visit 3 it was 505.6 ± 165.8 μm^2^; in visit 4 it was 488.5 ± 160.1 μm^2^; and in visit 5, it measured 475.2 ± 156.1 μm^2^. There was no statistically significant effect of the treatment with ranibizumab or aflibercept on the DCP FAZ area change (between-subjects effects *p* = 0.277).

Post hoc pairwise comparisons revealed that, at each subsequent study visit, the DCP FAZ area was smaller compared to the baseline (all *p* < 0.001). In addition, the DCP FAZ area at the last study timepoint was significantly smaller compared to any of the study visits timepoints (post hoc pairwise comparisons, all *p* < 0.001).

**Figure 2:**
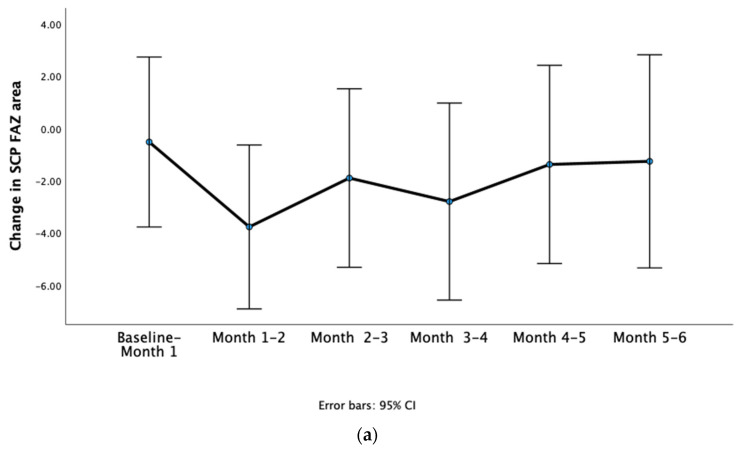

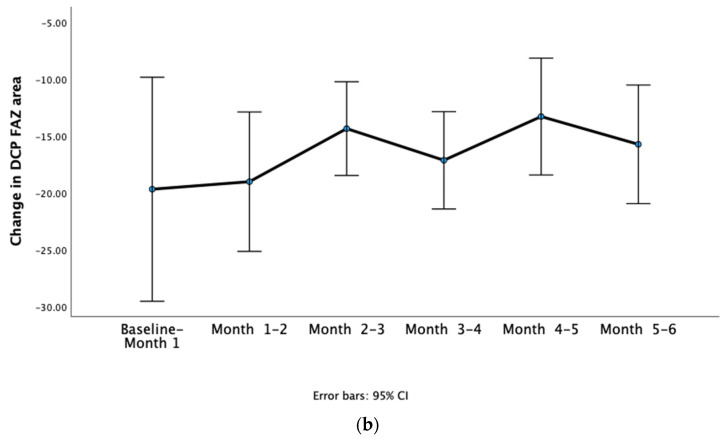
Line graphs of the change in superficial capillary plexus fovea avascular zone area (SCP FAZ, (**a**)) area and deep capillary plexus fovea avascular zone area (DCP FAZ, (**b**)) from baseline to month 1, from month 1 to month 2, from month 2 to month 3, from month 3 to month 4, from month 4 to month 5, and finally, from month 5 to month 6.

Then, the difference between FAZ area measurements in consecutive visits was calculated. As shown graphically in Figure 2a,b, there was variability in the amount of change in the SCP FAZ area and DCP FAZ area occurring every month.

There was no significant difference in the change in SCP FAZ area between consecutive monthly visits (repeated measures ANOVA *p* = 0.791). The change in SCP FAZ area was measured at 0.5 ± 11.5 μm^2^ from the baseline visit to visit 1; 3.8 ± 11.0 μm^2^ from visit 1 to visit 2; 1.9 ± 12.0 μm^2^ from visit 2 to visit 3; 2.8 ± 13.3 μm^2^ from visit 3 to visit 4; 1.4 ± 13.3 μm^2^ from visit 4 to visit 5; and 1.3 ± 14.4 μm^2^ from visit 5 to visit 6.

Furthermore, there was no significant difference in the change in DCP FAZ area between the consecutive monthly visits (repeated measures ANOVA *p* = 0.368). The change in DCP FAZ area was measured at 19.7 ± 34.6 μm^2^ from the baseline visit to visit 1, 19.0 ± 21.5 μm^2^ from visit 1 to visit 2; 14.3 ± 14.5 μm^2^ from visit 2 to visit 3; 17.1 ± 15.0 μm^2^ from visit 3 to visit 4; 13.3 ± 18.1 μm^2^ from visit 4 to visit 5; and 15.6 ± 18.3 μm^2^ from visit 5 to visit 6.

Then, correlations between BCVA, CRT, and FAZ area measurements were estimated. At baseline, there was no correlation between the SCP FAZ area or DCP FAZ area or CRT and BCVA (*p* = 0.108, *p* = 0.337 and *p* = 0.184, respectively). A weak correlation existed between CRT and SCP FAZ area at baseline (r = 0.311, *p* = 0.028).

Interestingly, there was no correlation between the SCP FAZ area and DCP FAZ area (*p* = 0.235) at baseline. In the final visit, after 6 intravitreal anti-VEGF injections, the only significant association was a weak correlation between the SCP FAZ area and DCP FAZ area (r = 0.315, *p* = 0.026). The SCP FAZ area was larger than the DCP FAZ area at all timepoints (*p* < 0.001).

Finally, we tested whether the size of the SCP FAZ area and DCP FAZ area at baseline correlates with the amount of BCVA gain with intravitreal treatment. There was no correlation between either the SCP FAZ area (*p* = 0.598) or DCP FAZ area (*p* = 0.768) and BCVA gain.

## 4. Discussion

In the present study, OCT angiography imaging was used to quantify the effects of anti-VEGF intravitreal injections on SCP and DCP FAZ area and explore the changes that occur over time with a fixed treatment regimen. This was studied in all participants with monthly measurements and monthly injections for a period of 6 months. Interestingly, our results suggest that the DCP FAZ area decreases with intravitreal injections of ranibizumab or aflibercept in the treatment naïve DME. In fact, the DCP FAZ area continued to decrease up to the 6 months visit. There was no effect of the choice of either ranibizumab or aflibercept detected in our cohort. There was also no significant change in the SCP FAZ area, while BCVA increased and CRT decreased with treatment, as expected.

The role of anti-VEGF drugs in the evolution of ischemia has been previously addressed with the use of FA. Indeed, it was speculated that treatment with anti-VEGF agents may in some cases even aggravate macula nonperfusion and ischemia [17]. The hypothesis that anti-VEGF drugs could lead to increasing areas of ischemia was extensively studied, analyzing data from the RISE and RIDE studies [18] as well as the RESTORE study [19]. Indeed, Campochiaro et al. suggested that ranibizumab slows the progression of ischemia [18]. The analysis of FA from the RESTORE study also concluded that repeated ranibizumab treatment was not associated with impaired macular perfusion. In fact, the authors urged physicians to offer therapy to patients with severe microangiopathy and advanced capillary dropout, based on their findings [19]. In their study, Michaelides et al. also reported no change in FAZ area in a group of patients treated with bevacizumab for DME [20]. Therefore, based on the analysis of FA images, anti-VEGF treatment may in fact improve macular ischemia and change the natural history of the disease.

Previous studies in the literature with OCT angiography explored the changes in FAZ with anti-VEGF treatment and reported either a decrease in FAZ area or no change. In their study, Falavarjani et al. measured FAZ area and vessel density before and after a single intravitreal anti-VEGF injection and no difference was detected [21]. Our group previously reported that SCP FAZ remains similar, whereas DCP FAZ area and DCP ischemia decreases with three aflibercept injections [22]. The superficial FAZ area showed improvement after 3 ranibizumab injections in the study by Hsieh et al. using external software to analyze OCT angiography images [23]. SCP FAZ remained unchanged in another study in DME patients receiving anti-VEGF injections, in line with our study [24]. In another recent report, Busch et al. quantified the FAZ area and retinal vascular area before and after aflibercept intravitreal injections in 23 eyes with DME [25], and in their patients, the FAZ area remained unchanged after treatment. Finally, Sorour et al. reported that the macular vessel density remained unchanged in their cohort of patients with diabetic macular edema and proliferative diabetic retinopathy [26]. These differences may be attributable to the segmentation algorithms and the differences in each measuring algorithm utilized by each imaging platform, the selection of the swept source or spectral domain OCT and the evolution that each of these platforms has undergone in recent years, and possibly the inherent ocular characteristics of each study subject.

Moreover, the amount of difference in the DCP FAZ area occurring every month with treatment did not significantly change over time. Interestingly, the DCP FAZ area continued to decrease and therefore at least some patients continue to benefit from ongoing treatment. The analysis of our data did not point to a specific timepoint where this change occurs primarily in all patients. There is variability, as shown also in the 95% confidence intervals, when looking at the DCP FAZ area change data.

Our study also found that the SCP FAZ area is smaller than DCP FAZ area, which is in line with previous reports. Notably, the primary variation observed over time was in the DCP FAZ area, while the SCP FAZ area remained relatively constant throughout the duration of the study. Hence, the resolution of edema primarily impacts the structure surrounding the deep capillary network. The administration of ranibizumab or aflibercept resulted in comparable alterations in the DCP FAZ area. Furthermore, we observed no correlation between the FAZ area measurements and BCVA gain or CRT improvement in our patients. This is not surprising, as there is a significant amount of variability in all of these parameters and the sample size is relatively small to accurately identify any possible associations. Larger studies with more patients can provide more power to detect such differences. Unlike OCT, it is generally accepted that there are likely no baseline OCT angiography biomarkers that directly correspond to the degree of improvement in anatomy [23]. Moreover, employing new methods to analyze the data on the FAZ area contained within the OCT angiography images could potentially reduce the extent of individual variability [27].

There are specific constraints on our study. An important factor to consider is that the retinal vasculature exhibits a three-dimensional configuration. Our approach involves analyzing the changes that occur with treatment by looking only at two slabs from the OCT angiography imaging. Therefore, we choose to only analyze a fraction of the information that is contained in the OCT angiography scans. This is naturally superior to looking at fluorescein angiography images, but examining the volume of the capillary plexi would be a more effective approach and would in fact provide a more accurate answer to the question of whether areas are truly re-perfused with treatment [28].

In addition, the presence of edema impacts the OCT angiography software algorithm’s capacity to segment the tissue. It is more common for OCT angiography segmentation algorithms to fail to recognize correctly the anatomic boundaries of each slab in the presence of cystic spaces in the retina, compared to the healthy macula. For this reason, we also looked at specific images to check for segmentation errors. Remarkably, the built-in algorithm exhibited an excellent performance and we did not observe any substantial deviations. A further limitation arises from the manual nature of the measurements conducted for SCP and DCP FAZ area sizes. This is counterbalanced by the fact that the mean of two measurements was documented for each timepoint, as well as by the fact that the two graders showed remarkable agreement and there was very satisfactory intraindividual repeatability. The FAZ area is inherently variable, particularly in the presence of intraretinal fluid. Nevertheless, previous studies have reported that the repeatability and reproducibility were excellent, in both normal eyes and in diabetics. However, it should be noted that there was greater variability in DCP FAZ area compared to the SCP FAZ area [29,30].

In conclusion, our study measured the change over time in the FAZ area in both the superficial and the deep capillary plexus of the retina. While no statistically significant difference was detected in the superficial capillary plexus FAZ area, deep-layer ischemia improved and the DCP FAZ area decreased with each injection. This effect was seen with both anti-VEGF drugs used; aflibercept and ranibizumab. Monthly measurements were conducted to more accurately describe the time series of changes and the impact in deep vasculature remained consistent. OCT angiography imaging can offer clinical insights for assessing and tracking the severity of deep macular ischemia and its evolution with anti-VEGF treatment.

## Figures and Tables

**Table 1:** Best corrected visual acuity (BCVA), central retina thickness (CRT), superficial capillary plexus foveal avascular zone area (SCP FAZ) and deep capillary plexus foveal avascular zone area (DCP FAZ) summary statistics in each study visit. Measurements are presented as mean ± standard deviation.

	Baseline	Month 1	Month 2	Month 3	Month 4	Month 5	Month 6	*p*-Value
BCVA (ETDRS letters)	40.8 ± 10.0	46.0 ± 9.1	47.8 ± 9.2	48.8 ± 8.3	49.4 ± 8.2	50.2 ± 7.9	52.1 ± 7.9	<0.001
CRT (μm)	295.6 ± 33.9	273.3 ± 32.6	262.3 ± 31.1	252.6 ± 30.6	248.0 ± 31.9	247.9 ± 29.7	253.0 ± 29.8	<0.001
SCP FAZ (μm^2^)	350.6 ± 79.5	350.1 ± 79.2	346.3 ± 77.0	344.4 ± 77.6	341.6 ± 77.1	340.2 ± 73.3	339.0 ± 71.3	0.132
DCP FAZ (μm^2^)	558.6 ± 199.0	539.0 ± 182.0	520.0 ± 169.4	505.6 ± 165.8	488.5 ± 160.1	475.2 ± 156.1	459.5 ± 156.1	<0.001

## Data Availability

Data are available upon reasonable request.
